# Supplementary material for: Somatic Evolution of a Germline Antibody Expands its Breadth to Neutralize Early SARS‐CoV‐2 Omicron Variants
Source: Adv Sci (Weinh). 2026 Jul 13:e76522. Online ahead of print. doi: 10.1002/advs.76522 (PMC13359428; doi:10.1002/advs.76522)
Supplement: Supplementary file 1 — Supporting File 1: advs76522‐sup‐0001‐SuppMat.docx. [file ADVS-9999-e76522-s002.docx]

**Data S1. BA1 neutralizing and non-neutralizing sequences**

**Table S1. X-ray data collection and refinement statistics**

| **Data collection** | SARS-CoV-2 wild-type RBD + HB148 + LY-CoV1404 | SARS-CoV-2 wild-type RBD + HB148-M4 + LY-CoV1404 | SARS-CoV-2 Omicron BA.1 RBD + HB148-M4 + LY-CoV1404 |
| --- | --- | --- | --- |
| Beamline | NSLS-II 17-ID-2 | SSRL BL12-1 | SSRL BL12-1 |
| Wavelength (Å) | 0.97934 | 0.97946 | 0.97946 |
| Space group | P1 | C 1 2 1 | C 1 2 1 |
| Unit cell parameters |  |  |  |
| a, b, c (Å) | 85.3, 86.1, 98.8 | 195.8, 88.7, 99.9 | 196.8, 88.0, 100.8 |
| α, β, γ (°) | 115.7, 89.7, 94.4 | 90, 112.0, 90 | 90, 110.3, 90 |
| Resolution (Å) ^a^ | 50.0-3.10 (3.15-3.10) | 50.0-2.60 (2.64-2.60) | 50.0-2.73 (2.78-2.73) |
| Unique reflections ^a^ | 45,996 (2,261) | 47,927 (2,367) | 42,283 (1,794) |
| Redundancy ^a^ | 2.0 (1.9) | 4.7 (3.1) | 5.5 (3.3) |
| Completeness (%) ^a^ | 97.8 (96.7) | 98.0 (97.7) | 96.5 (82.7) |
| <I/σ_I_> ^a^ | 4.3 (1.0) | 15.4 (1.2) | 14.7 (1.3) |
| *R*_sym_^b^ (%) ^a^ | 17.6 (65.3) | 11.5 (>100) | 15.6 (>100) |
| *R*_pim_^b^ (%) ^a^ | 15.2 (58.4) | 5.7 (69.5) | 6.8 (55.9) |
| CC_1/2_^c^ (%) ^a^ | 99.6 (40.3) | 98.7 (58.8) | 98.3 (68.8) |
| **Refinement statistics** |  |  |  |
| Resolution (Å) | 48.8-3.10 | 36.1-2.60 | 40.8-2.73 |
| Reflections (work) | 39,220 | 42,405 | 34,641 |
| Reflections (test) | 1,841 | 2,109 | 2,000 |
| *R*_cryst_^d^ / *R*_free_^e^ (%) | 21.3/25.9 | 19.3/24.3 | 20.0/25.3 |
| Copies of Fab/RBD per ASU | 2 | 1 | 1 |
| No. of atoms | 15,954 | 8,159 | 8,052 |
| Fab | 12,828 | 6,420 | 6,438 |
| RBD | 3,116 | 1,558 | 1,522 |
| Ligands ^f^ | 10 | 17 | 42 |
| Water | 0 | 156 | 50 |
| Average *B-*values (Å^2^) | 69 | 50 | 50 |
| Fab | 70 | 51 | 51 |
| RBD | 65 | 49 | 48 |
| Ligands ^f^ | 75 | 51 | 54 |
| Water | - | 40 | 31 |
| Wilson *B*-value (Å^2^) | 67 | 44 | 43 |
| **RMSD from ideal geometry** |  |  |  |
| Bond length (Å) | 0.002 | 0.002 | 0.002 |
| Bond angle (^o^) | 0.50 | 0.55 | 0.52 |
| **Ramachandran statistics (%) ^g^** |  |  |  |
| Favored | 96.95 | 97.58 | 97.18 |
| Outliers | 0.05 | 0.00 | 0.00 |
| **PDB code** | 9ZBW | 9ZBX | 9ZBY |

^a^ Numbers in parentheses refer to the highest resolution shell.

^b^ *R*_sym_ = Σ*_hkl_* Σ*_i_* | I*_hkl,i_* - <I*_hkl_*> | / Σ*_hkl_* Σ*_i_* I*_hkl,i_* and R*_pim_* = Σ*_hkl_* (1/(n-1))^1/2^ Σ*_i_* | I*_hkl,i_* - <I*_hkl_*> | / Σ*_hkl_* Σ*_i_* I*_hkl,i_*, where I*_hkl,i_* is the scaled intensity of the i^th^ measurement of reflection h, k, l, <I*_hkl_*> is the average intensity for that reflection, and *n* is the redundancy.

^c^ CC_1/2_ = Pearson correlation coefficient between two random half datasets.

*^d^ R*_cryst_ = Σ*_hkl_* | *F*_o_ - *F*_c_ | / Σ*_hkl_* | *F*_o_ | x 100, where *F*_o_ and *F*_c_ are the observed and calculated structure factors, respectively.

^e^ *R*_free_ was calculated as for *R*_cryst_, but on a test set comprising ~4.7%-5.8% of the data excluded from refinement.

^f^ Bound ligands are phosphate, Tris(hydroxymethyl)aminomethane, and ethylene glycol molecules.

^g^ From MolProbity.^1^

**Table S2. NT50 for four IGHV3-53 antibodies and their M4 variants**

| **NT_50_ (μg/mL)** | **Wild-type strain** | **Alpha strain** | **Beta strain** | **Gamma strain** | **Delta strain** | **BA.1 strain** | **BA.4/5 strain** | **XBB.1.5 strain** |
| --- | --- | --- | --- | --- | --- | --- | --- | --- |
| **C1A-F10 WT** | 0.084 | 6.727 | 23.550 | 22.920 | 0.824 | >100 | >100 | >100 |
| **C1A-F10 M4** | 0.032 | 0.117 | 42.920 | 69.340 | 0.161 | >100 | >100 | >100 |
| **P5A-3C8 WT** | 0.032 | >100 | >100 | >100 | 0.009 | >100 | >100 | >100 |
| **P5A-3C8 M4** | 0.034 | 0.091 | 0.339 | 6.391 | 0.012 | >100 | >100 | >100 |
| **BG4-25 WT** | 0.074 | 0.012 | 1.295 | 0.156 | 0.026 | >100 | >100 | >100 |
| **BG4-25 M4** | 0.008 | 0.002 | 0.013 | 0.031 | 0.033 | 0.047 | 0.101 | >100 |
| **LY-CoV488 WT** | 0.739 | 8.695 | >100 | >100 | 0.924 | >100 | >100 | >100 |
| **LY-CoV488 M4** | 0.097 | 0.088 | 0.524 | 0.413 | 0.144 | 2.606 | 0.128 | >100 |

**
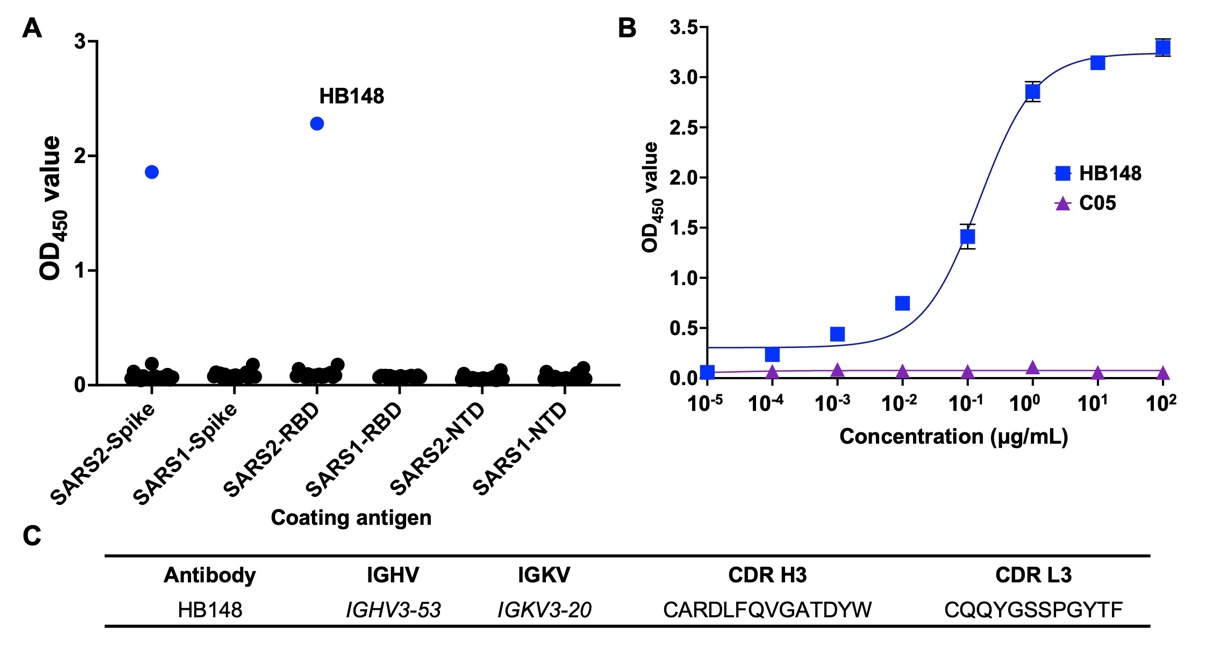
**

**Figure S1. Identification of human monoclonal antibodies to SARS-CoV-2 RBD. (A)** The binding activity of 24 human monoclonal antibodies against full spike, RBD, NTD protein from SARS-CoV-1 and SARS-CoV-2 was measured by ELISA. **(B)** The binding affinity of HB148 (blue) IgG against SARS-CoV-2 RBD was measured by ELISA. C05 is an influenza hemagglutinin antibody and serves as a negative control here.^2^ Data are representative of two independent experiments performed in technical duplicate. Error bars represent standard deviation. **(C)** Sequence information of antibody HB148 with heavy chain and light chain gene family and CDR3 amino acid sequence.


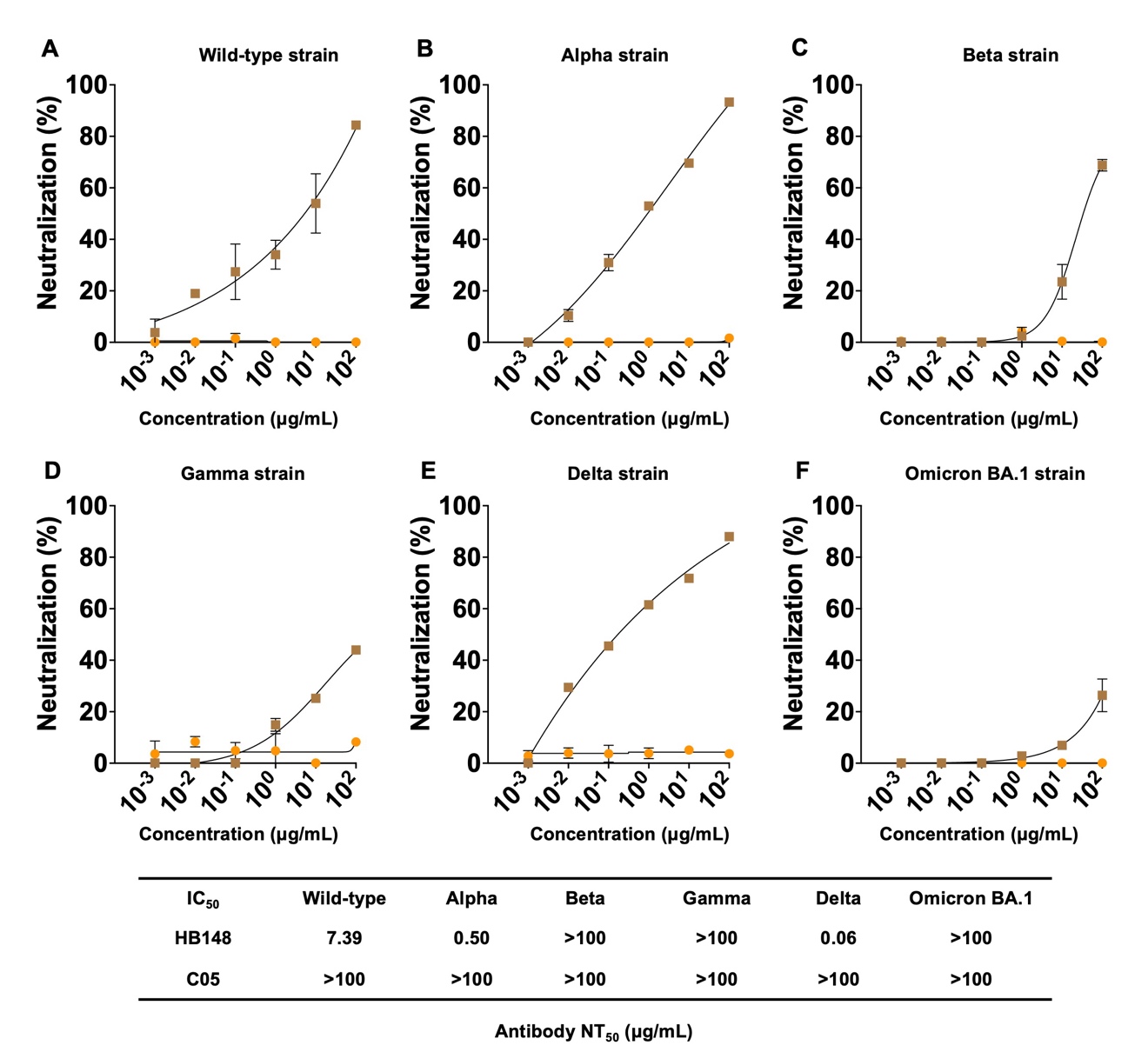


**Figure S2. Pseudovirus neutralizing ability of HB148 against different SARS-CoV-2 variants.** Neutralization activity of antibody HB148 was determined by SARS-CoV-2 pseudovirus neutralization assay against **(A)** Wild-type strain, **(B)** Alpha strain, **(C)** Beta strain, **(D)** Gamma strain, **(E)** Delta strain and **(F)** Omicron BA.1 strain. Their estimated NT_50_ values are indicated. C05 is an influenza hemagglutinin antibody and serves as a negative control here.^2^ Data points represent the mean of two technical replicates from each of two independent experiments; the line indicates the mean of the two independent experiments. Error bars represent standard deviation.

**
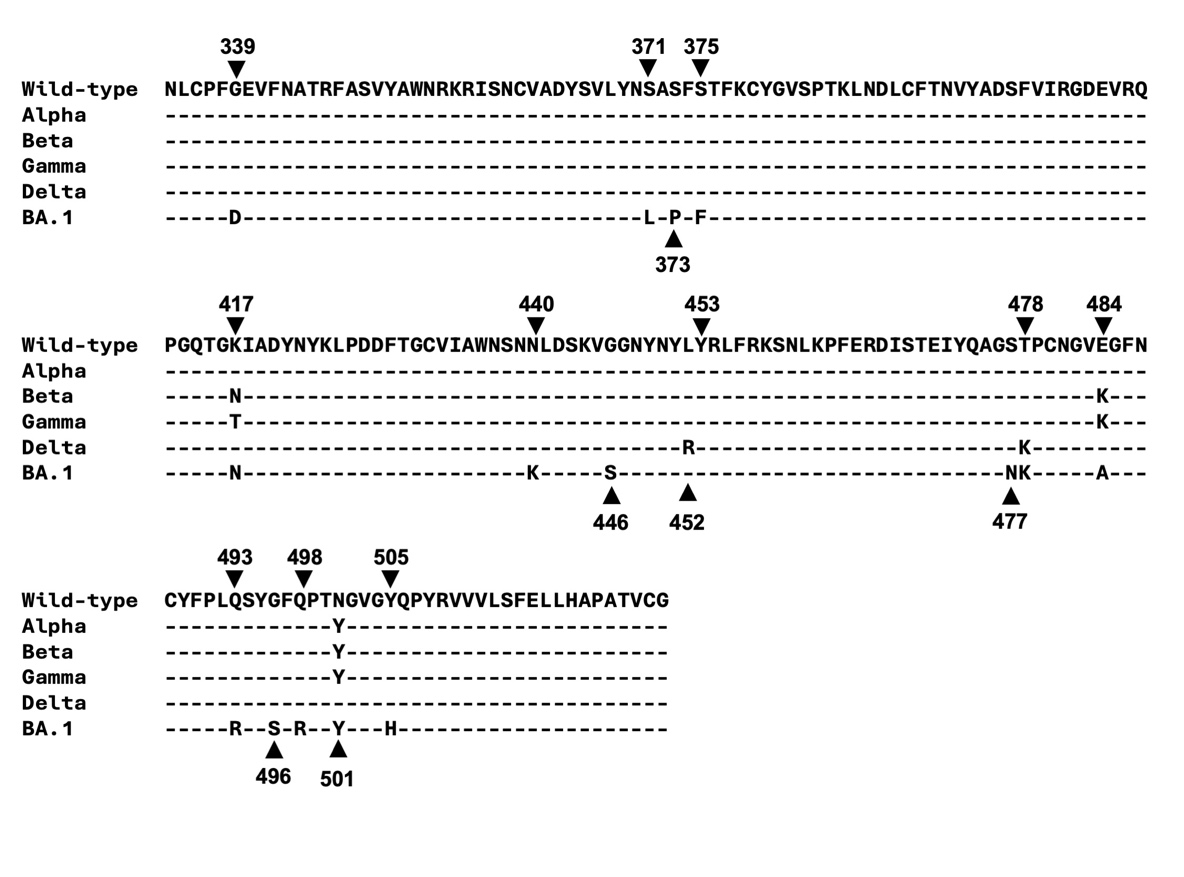
**

**Figure S3. Sequence alignment of RBDs from different SARS-CoV-2 variants.** Sequence alignment of RBD sequences from different SARS-CoV-2 variants was performed using MAFFT.^3^ Amino acid substitutions relative to the wild-type RBD are indicated, and triangle markers denote the positions of these mutations.

**
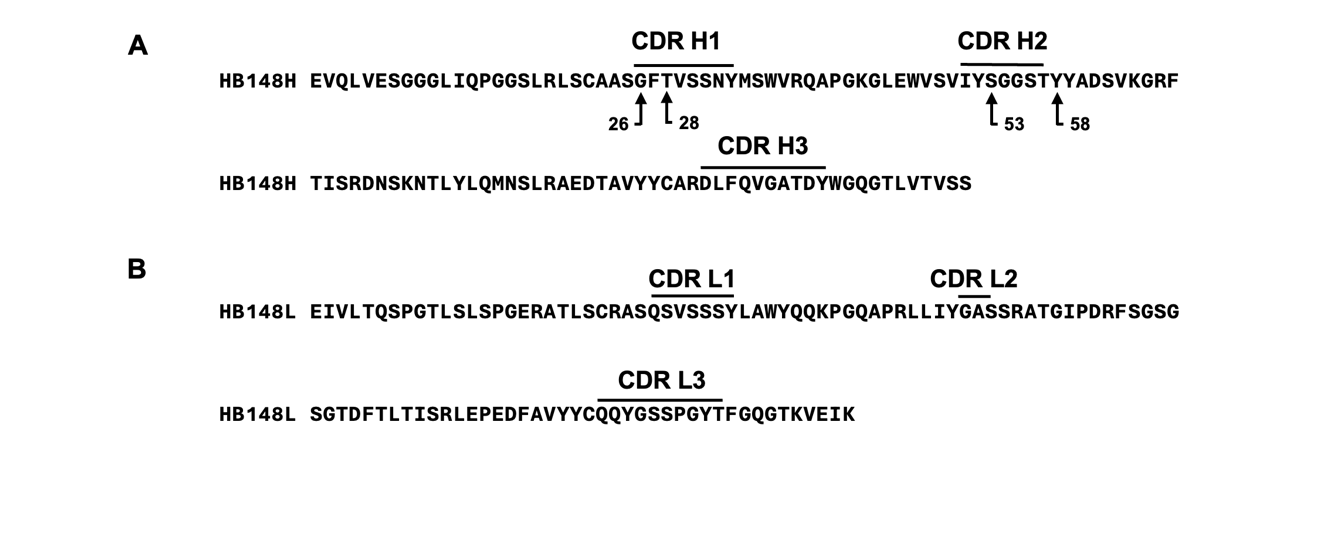
**

**Figure S4. Amino acid sequence information for antibody HB148 with heavy chain and light chain CDR regions highlighted.** CDR H1, CDR H2, CDR H3, and four potential somatic hypermutations in the HB148 heavy chain **(A)**, as well as CDR L1, CDR L2, and CDR L3 in the HB148 light chain **(B)**, were identified using IMGT.^4^


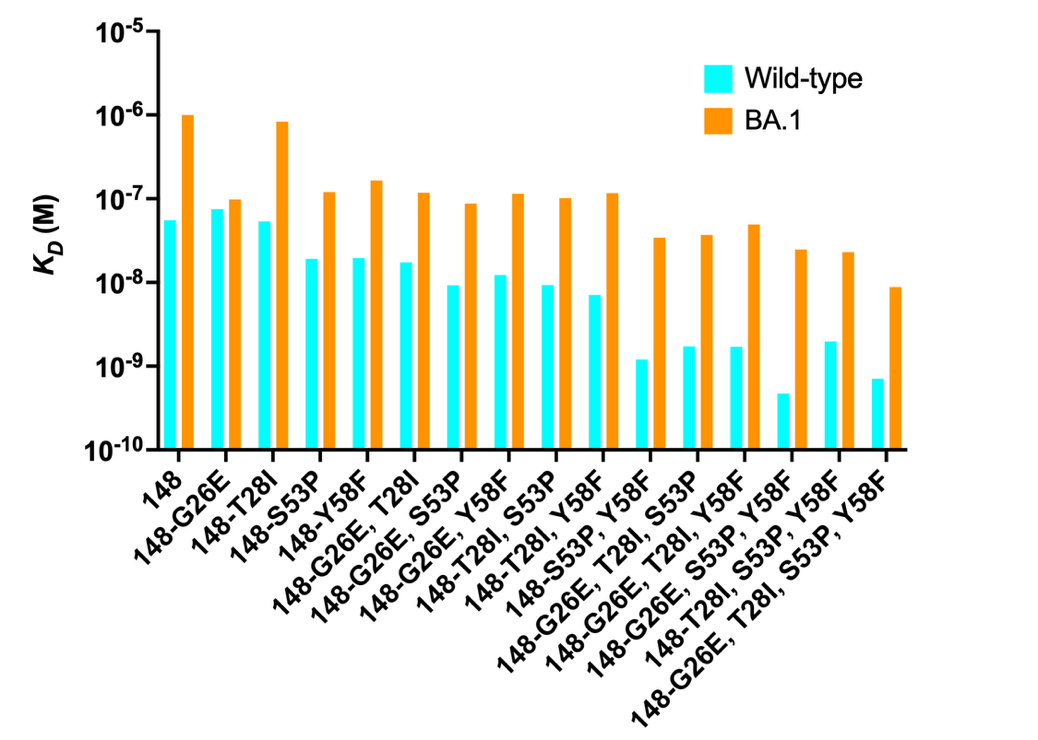


**Figure S5. Binding affinity of HB148 WT and mutants against wild-type and Omicron BA.1 RBD.** Binding kinetics of HB148 antibodies with different mutation combinations against wild-type RBD and Omicron BA.1 RBD were measured by biolayer interferometry (BLI). Binding affinities are reported as equilibrium dissociation constants (Kᴅ, M) on a logarithmic scale.


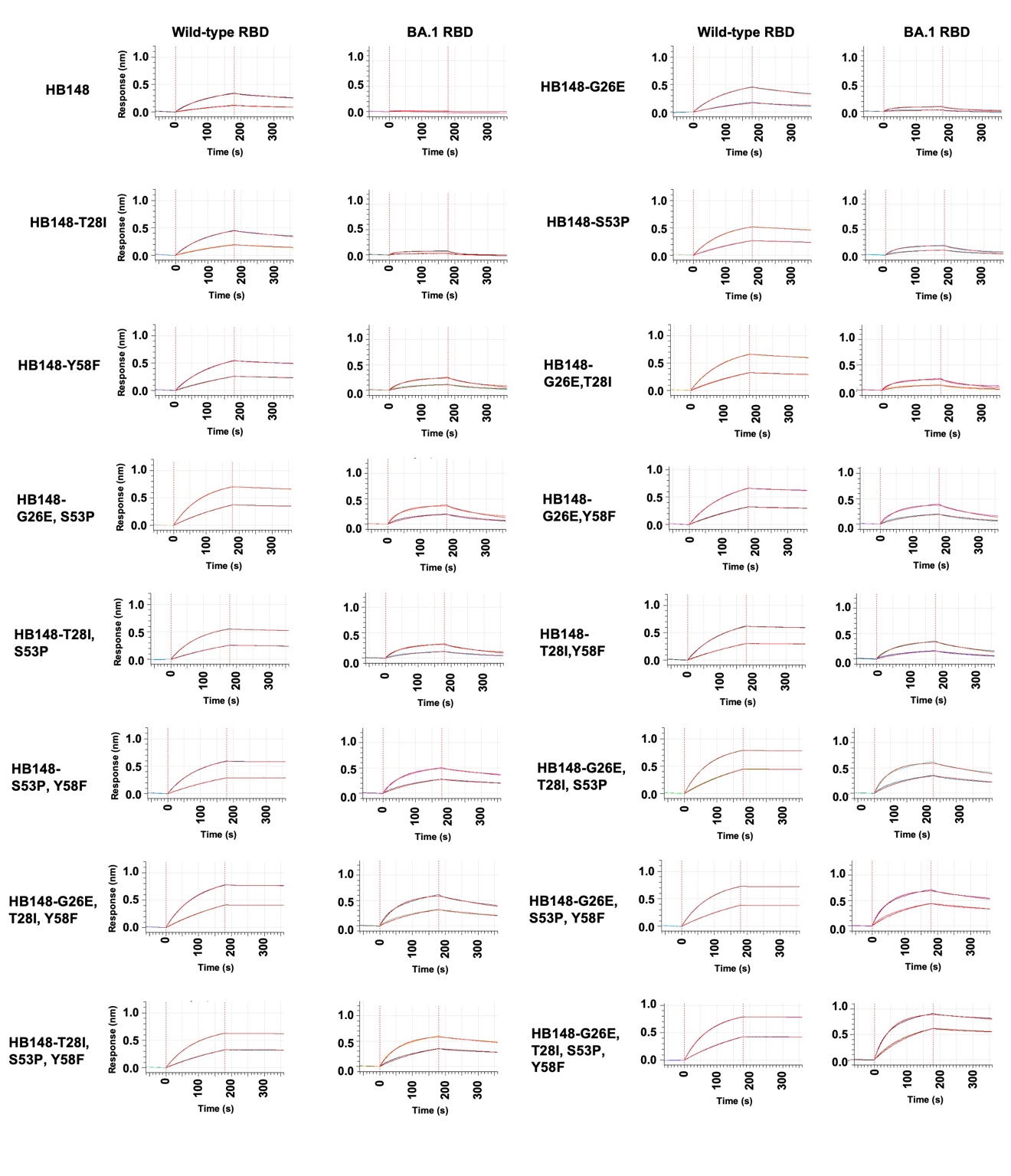


**Figure S6. Binding affinity of HB148 WT and mutants against wild-type and Omicron BA.1 RBD.** Binding kinetics of HB148 antibodies with different mutation combinations against wild-type RBD and Omicron BA.1 RBD were measured by biolayer interferometry (BLI). Y-axis represents the response.


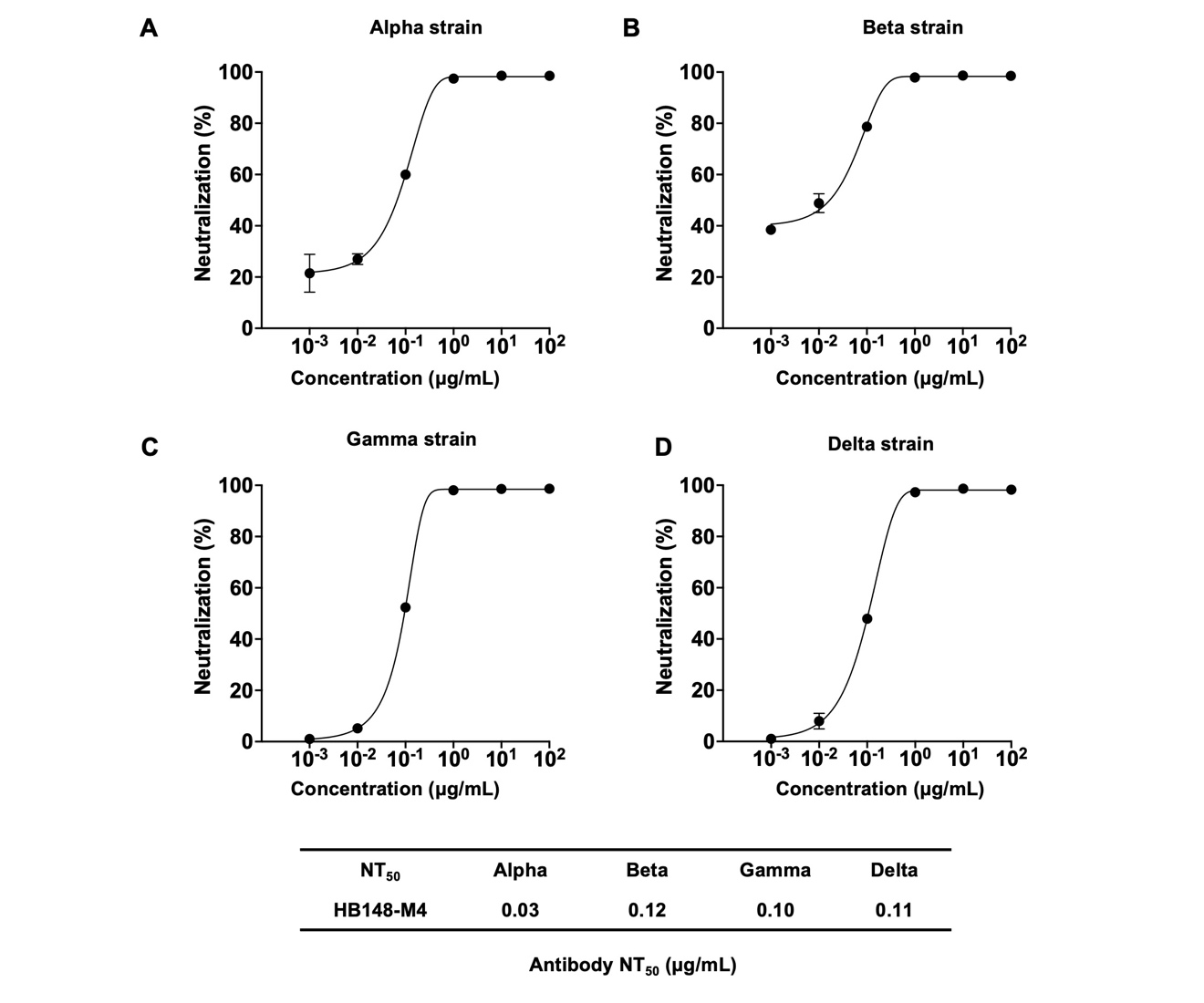


**Figure S7. Antibody neutralization of HB148-M4 against SARS-CoV-2 variants by sVNT.** Neutralization titer 50 (NT_50_) of antibody HB148-M4 against **(A)** Alpha strain, **(B)** Beta strain, **(C)** Gamma strain, **(D)** Delta strain were measured by surrogate virus neutralization test (sVNT). Their NT_50_ values are indicated. Data points represent the mean of two technical replicates from each of two independent experiments; the line indicates the mean of the two independent experiments. Error bars represent standard deviation.


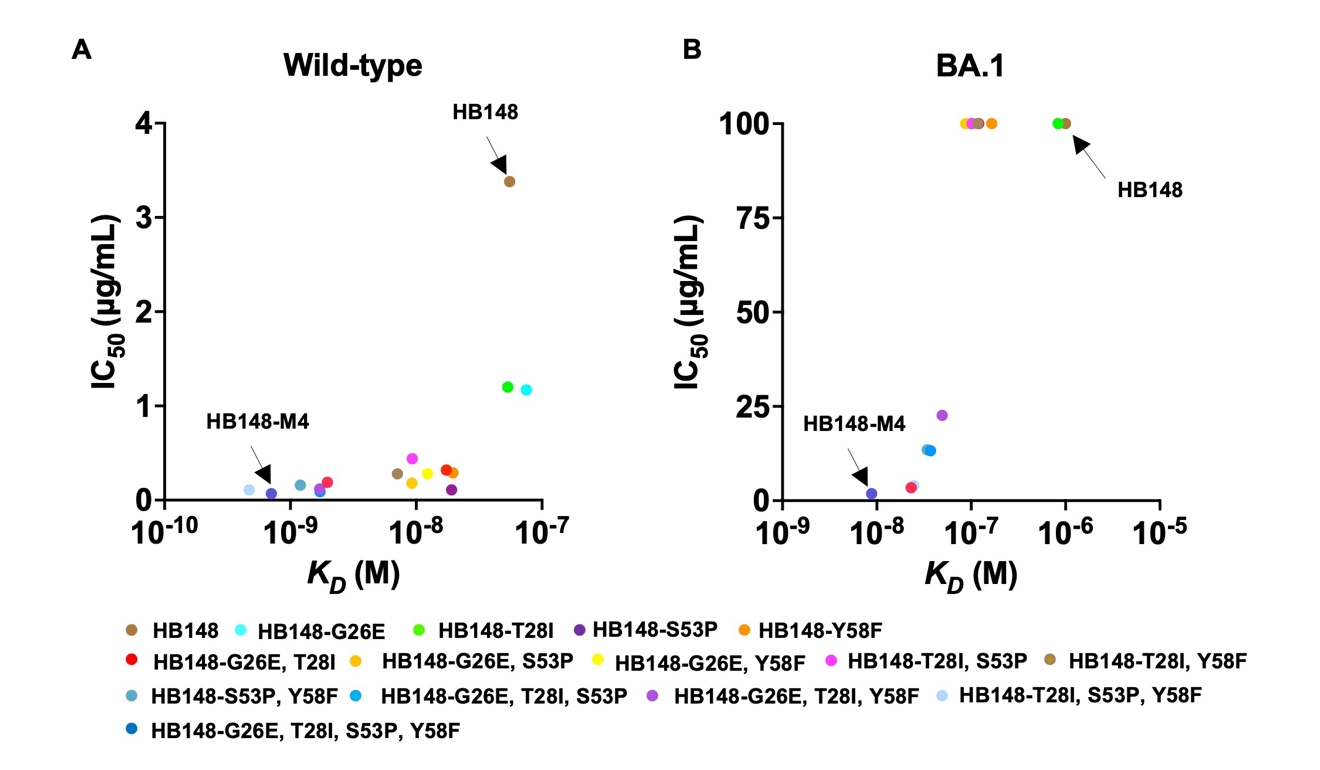


**Figure S8. Correlation between binding affinity (K_D_, M) and neutralization potency (IC_50_) of HB148 variants**. Scatter plots represent the relationship between the equilibrium dissociation constant (**K_D_, M**) and the half-maximal inhibitory concentration (**IC_50_**, µg/mL) for the parental HB148 antibody and its mutational derivatives. Data are shown for the **(A)** Wild-type strain and **(B)** Omicron BA.1 variant.

**
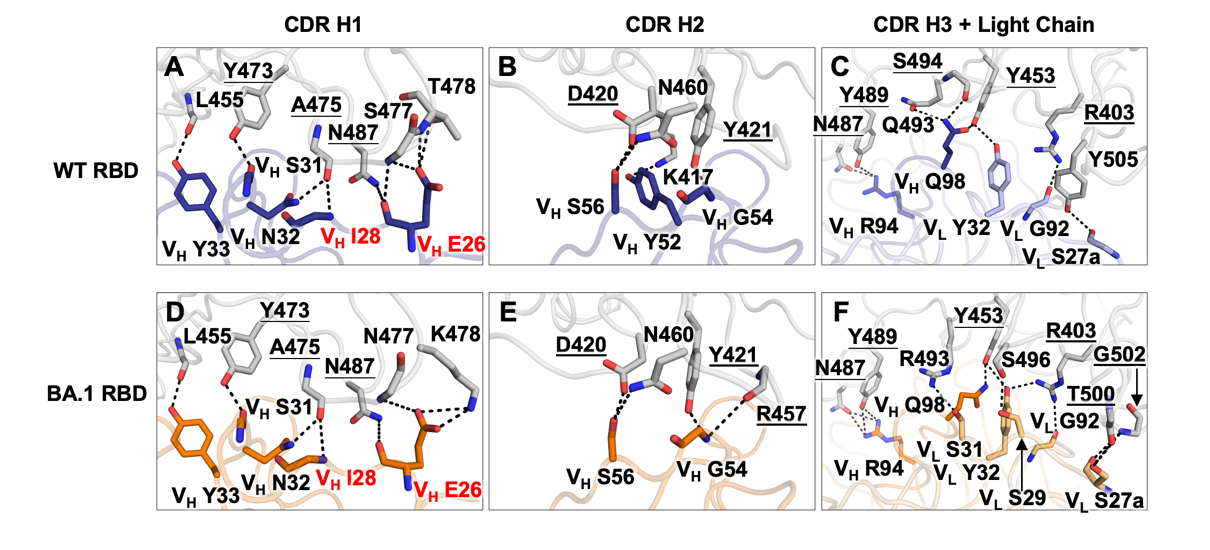
**

**Figure S9. Crystal structure analysis of HB148-M4 with wild-type RBD and BA.1 RBD.** Detailed molecular interactions (hydrogen bonds and salt bridges) of wild-type and BA.1 RBDs (grey backbone) with HB148-M4 are shown with V_H_ G26E and T28I labeled in red. Conserved epitope residues across VOCs are underlined.


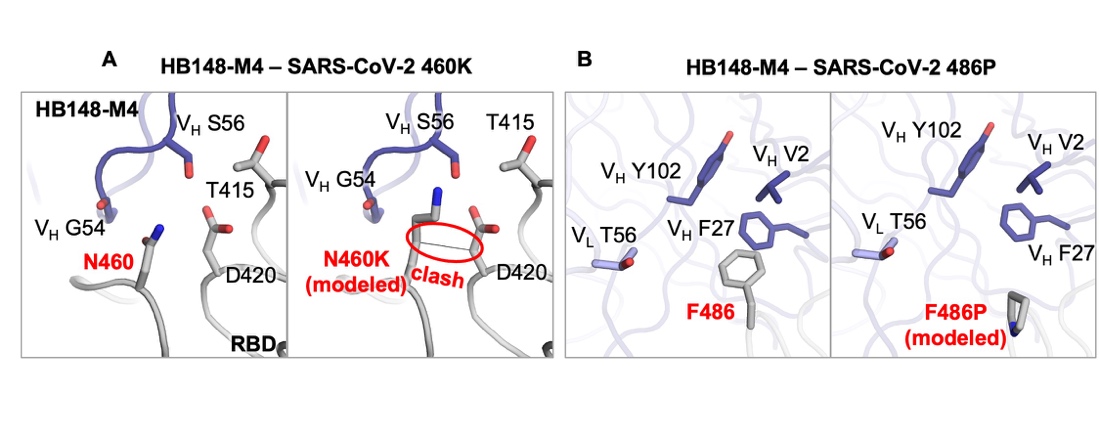


**Figure S10. Structural basis for the evasion of HB148-M4 by Omicron XBB.1.5 mutations. (A-B)** Close-up views of the binding interface at RBD residues 460 **(A)** and 486 **(B)**. The RBD is shown in grey, with the HB148-M4 heavy and light chains colored dark and light blue, respectively. Left panels illustrate the wild-type interface; right panels show modeled mutations.


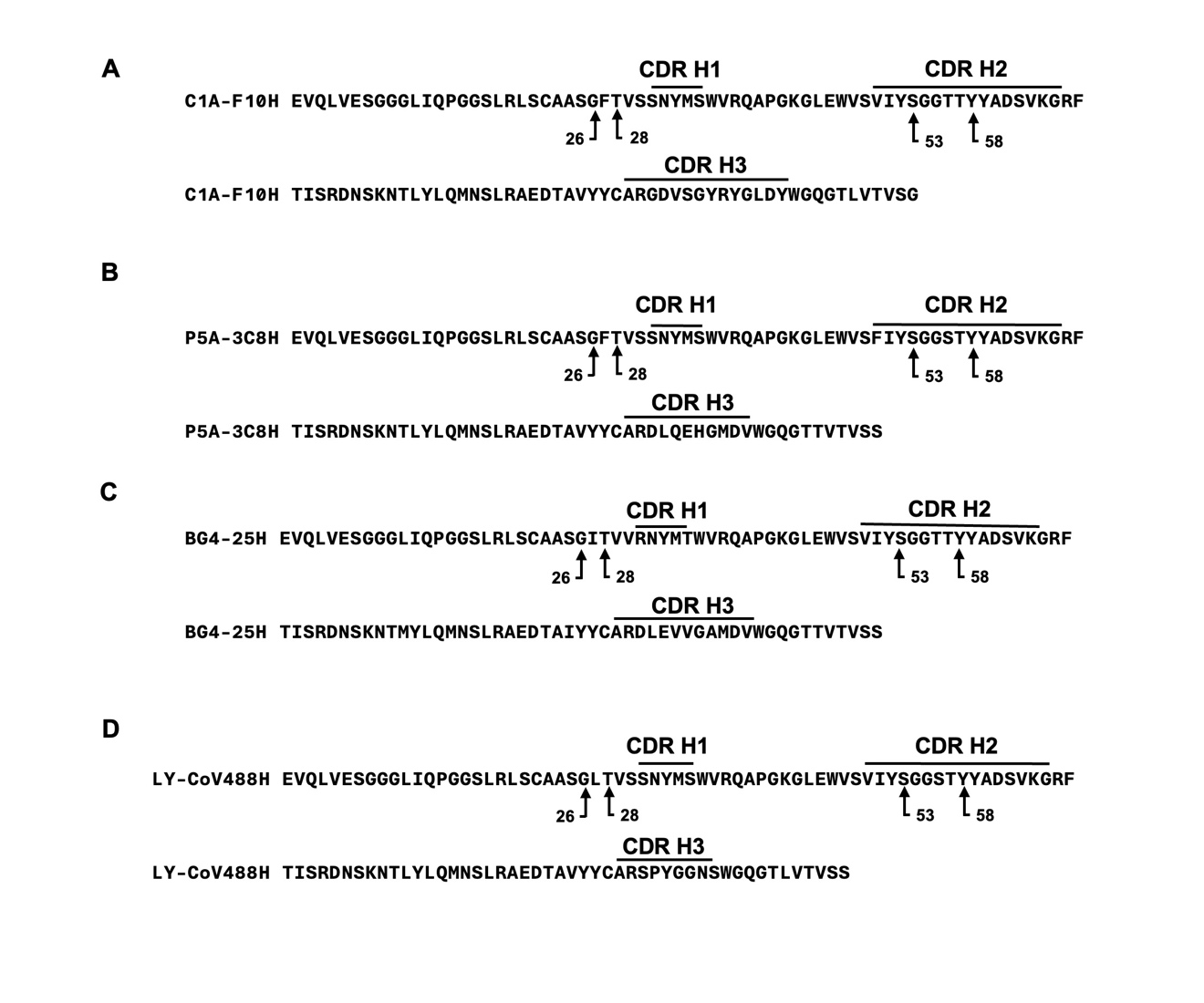


**Figure S11. Amino acid sequence information for antibody HB148 with heavy chain CDR regions highlighted.** CDR H1, CDR H2, CDR H3, and four potential somatic hypermutations in the C1A-F10^5^ **(A),** P5A-3C8^6^ **(B),** BG4-25^7^ **(C)** and LY-CoV488^8^ **(D)** heavy chains were identified using IMGT.^4^


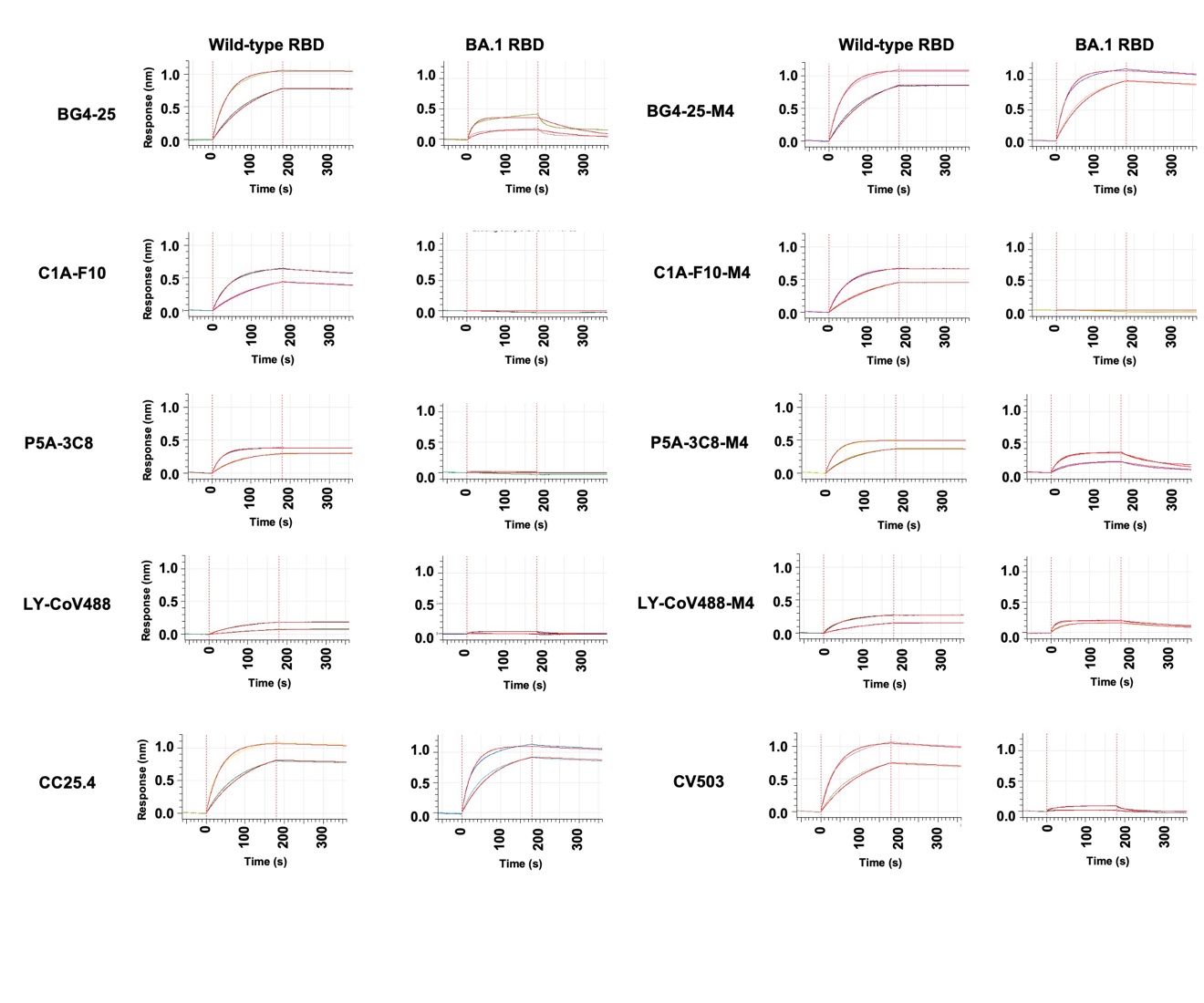


**Figure S12. Binding affinity of IGHV3-53 and M4 version antibodies against wild-type and Omicron BA.1 RBD.** Binding kinetics of IGHV3-53 antibodies and with M4 versions against wild-type RBD and Omicron BA.1 RBD were measured by biolayer interferometry (BLI). Y-axis represents the response.


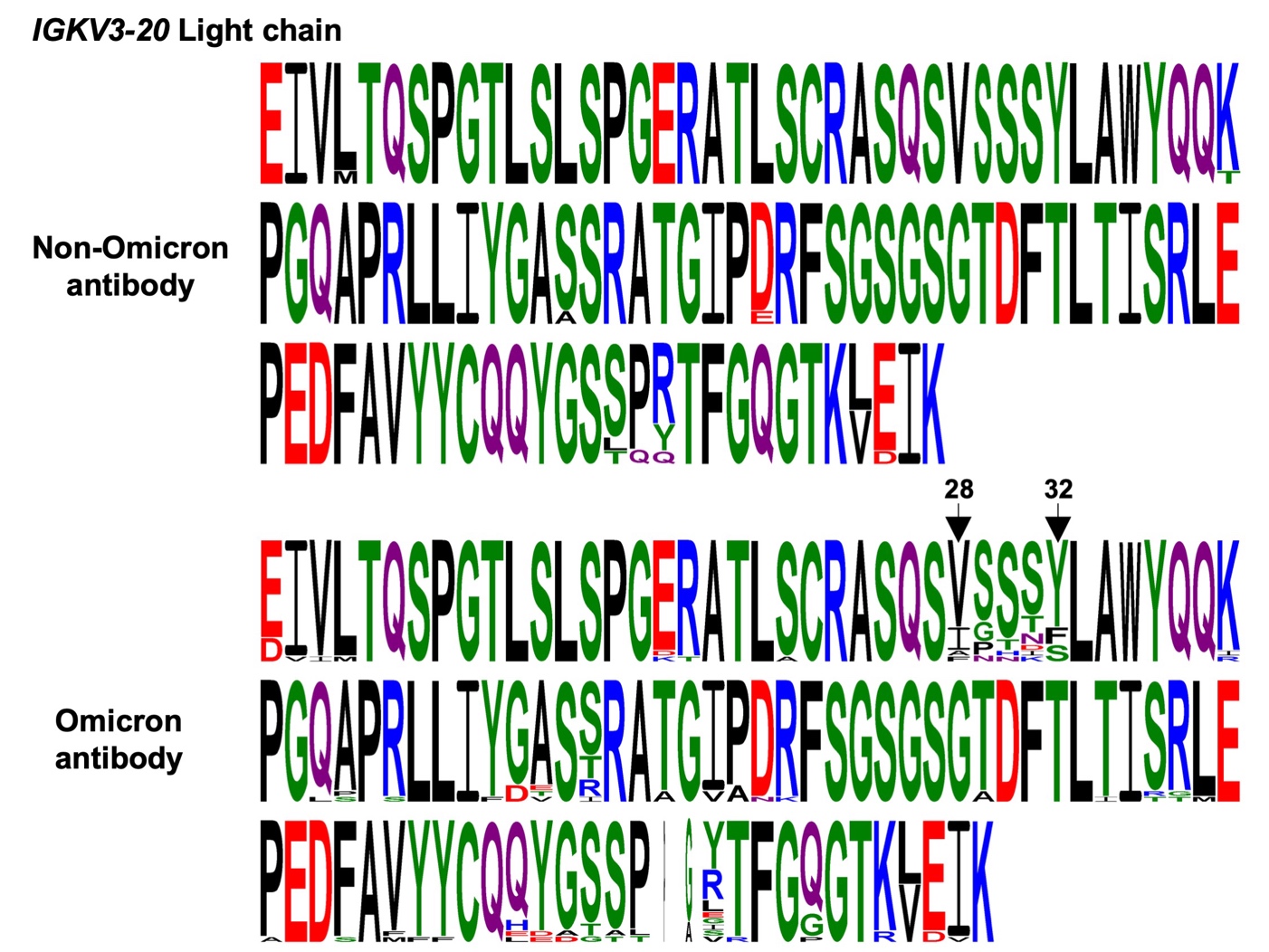


**Figure S13. *IGKV3-20* sequence alignment between Non-Omincron and Omicron neutralizing antibody.** IGKV3-20 sequence alignment between non-Omicron neutralizing antibodies and Omicron neutralizing antibodies. Sequences are downloaded from the Coronavirus Antibody Database (CoV-AbDab). Sequence logo has been plotted by WebLogo (<https://weblogo.berkeley.edu/logo.cgi>).

**SUPPLEMENTAL REFERENCES**

1 Williams, C. J. *et al.* MolProbity: More and better reference data for improved all-atom structure validation. *Protein Sci* **27**, 293-315 (2018). <https://doi.org/10.1002/pro.3330>

2 Ekiert, D. C. *et al.* Cross-neutralization of influenza A viruses mediated by a single antibody loop. *Nature* **489**, 526-532 (2012). <https://doi.org/10.1038/nature11414>

3 Katoh, K. & Standley, D. M. MAFFT multiple sequence alignment software version 7: improvements in performance and usability. *Mol Biol Evol* **30**, 772-780 (2013). <https://doi.org/10.1093/molbev/mst010>

4 Lefranc, M. P. *et al.* IMGT, the international ImMunoGeneTics information system. *Nucleic Acids Res* **37**, D1006-1012 (2009). <https://doi.org/10.1093/nar/gkn838>

5 Clark, S. A. *et al.* SARS-CoV-2 evolution in an immunocompromised host reveals shared neutralization escape mechanisms. *Cell* **184**, 2605-2617 e2618 (2021). <https://doi.org/10.1016/j.cell.2021.03.027>

6 Zhang, Q. *et al.* Potent and protective IGHV3-53/3-66 public antibodies and their shared escape mutant on the spike of SARS-CoV-2. *Nat Commun* **12**, 4210 (2021). <https://doi.org/10.1038/s41467-021-24514-w>

7 Scheid, J. F. *et al.* B cell genomics behind cross-neutralization of SARS-CoV-2 variants and SARS-CoV. *Cell* **184**, 3205-3221.e3224 (2021). <https://doi.org/10.1016/j.cell.2021.04.032>

8 Jones, B. E. *et al.* The neutralizing antibody, LY-CoV555, protects against SARS-CoV-2 infection in nonhuman primates. *Sci Transl Med* **13** (2021). <https://doi.org/10.1126/scitranslmed.abf1906>
