# Supplementary figures and images for: Somatic Evolution of a Germline Antibody Expands its Breadth to Neutralize Early SARS‐CoV‐2 Omicron Variants
Source: Adv Sci (Weinh). 2026 Jul 13:e76522. Online ahead of print. doi: 10.1002/advs.76522 (PMC13359428; doi:10.1002/advs.76522)

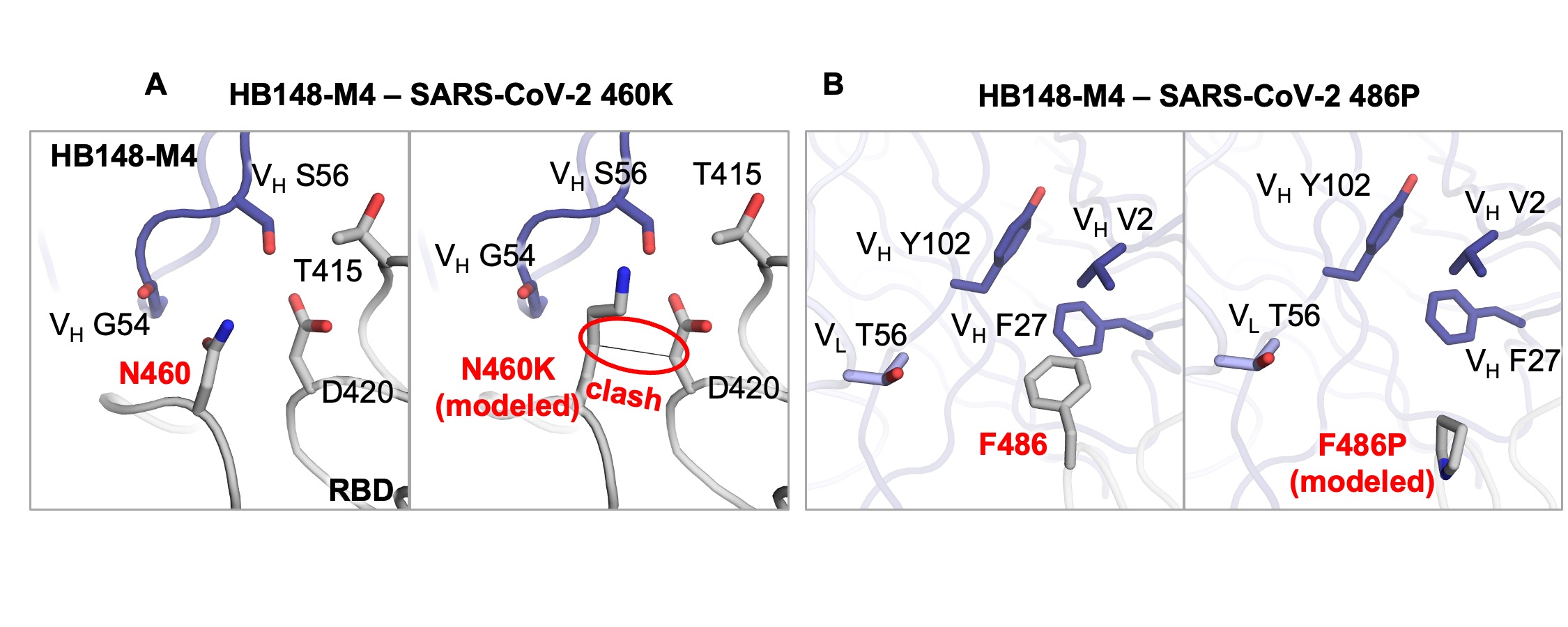

Supplement: Supplementary file 2 — Supporting File 2: advs76522‐sup‐0002‐FigureS1‐S13.zip. [file ADVS-9999-e76522-s001.zip › Figure S10_148_M4_N460_F486.jpg]

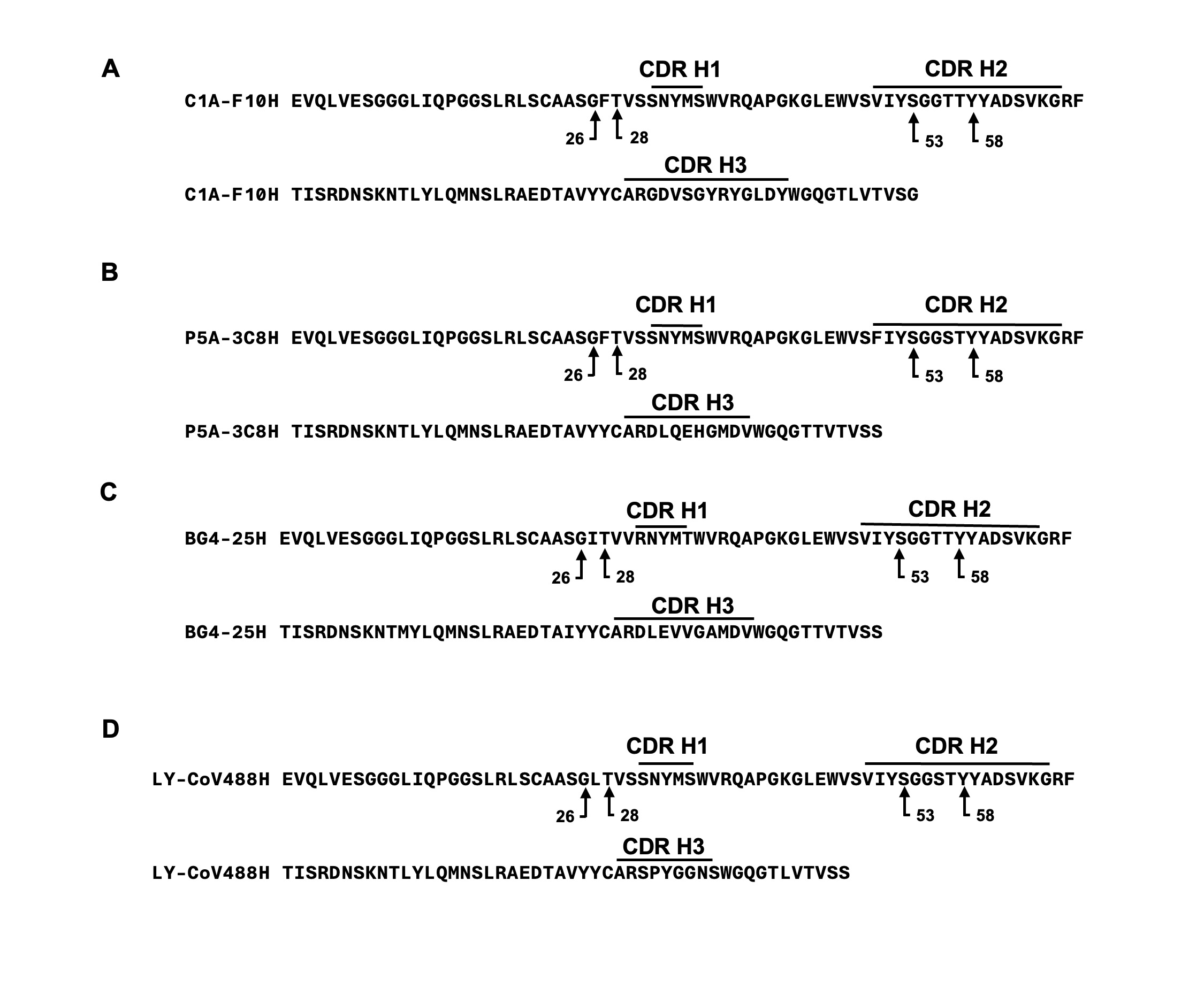

Supplement: Supplementary file 2 — Supporting File 2: advs76522‐sup‐0002‐FigureS1‐S13.zip. [file ADVS-9999-e76522-s001.zip › Figure S11_3-53 Ab.jpg]

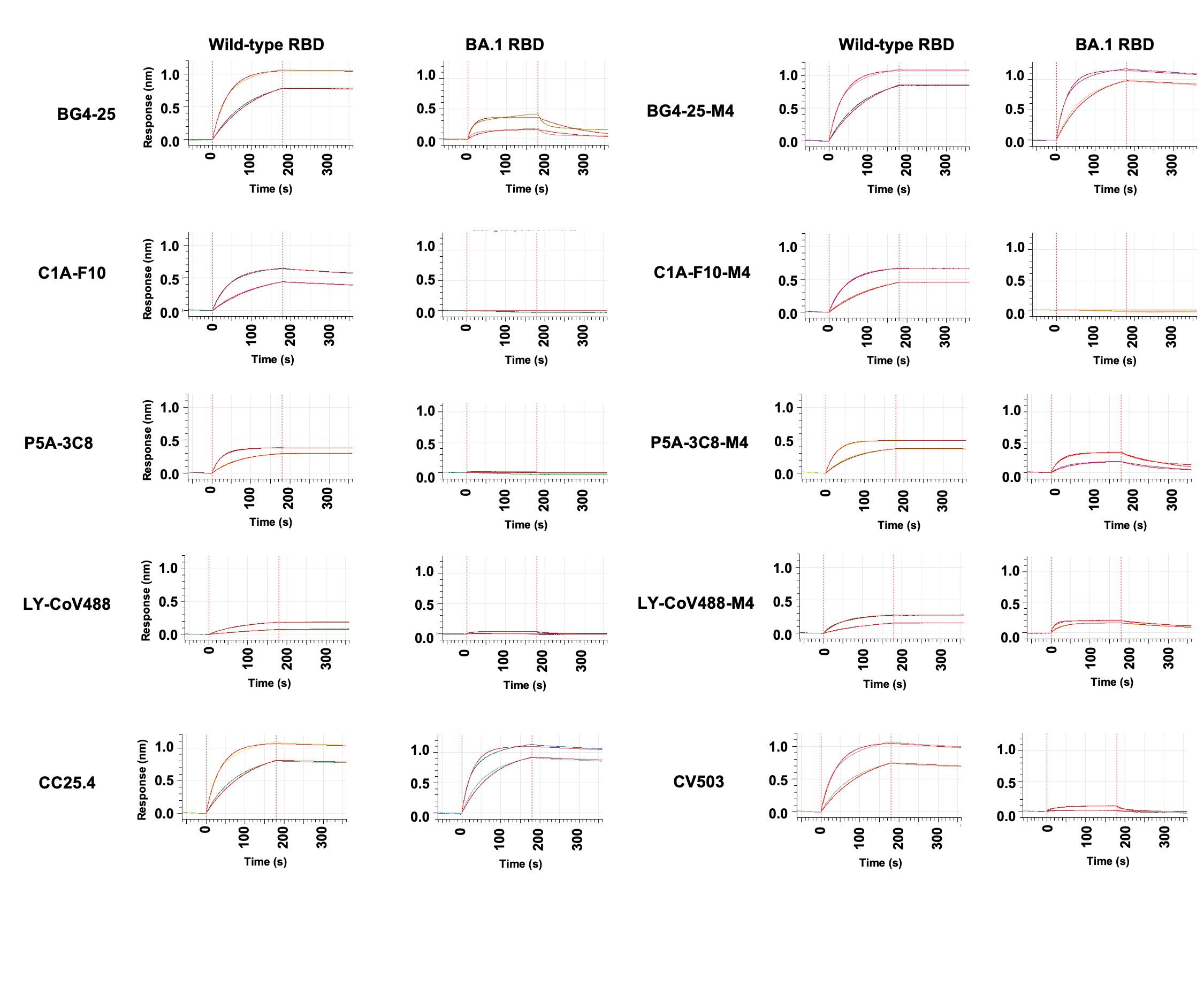

Supplement: Supplementary file 2 — Supporting File 2: advs76522‐sup‐0002‐FigureS1‐S13.zip. [file ADVS-9999-e76522-s001.zip › Figure S12-BLI-3-53.jpg]

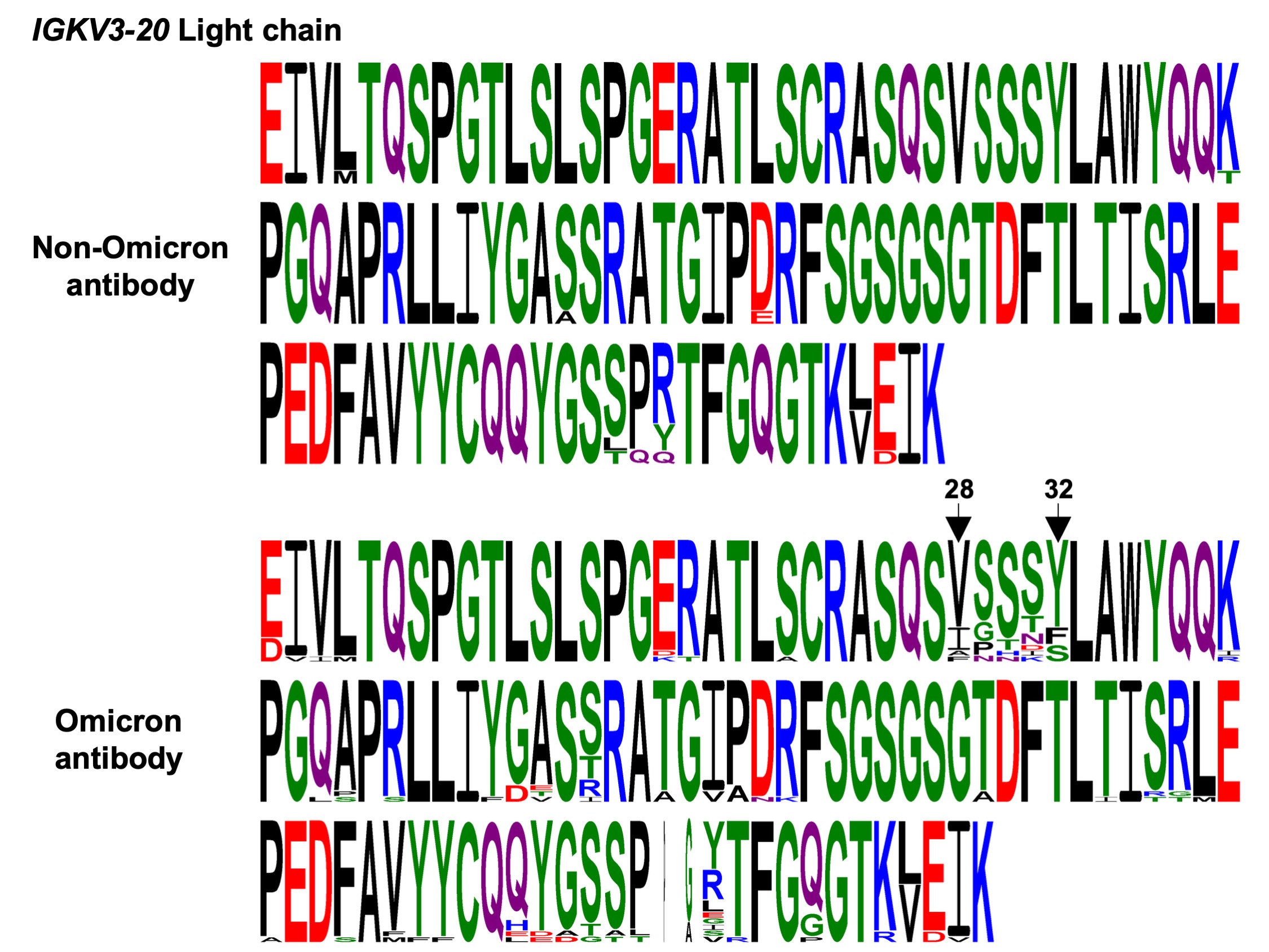

Supplement: Supplementary file 2 — Supporting File 2: advs76522‐sup‐0002‐FigureS1‐S13.zip. [file ADVS-9999-e76522-s001.zip › Figure S13_seqlog light chain and CDR3.jpg]

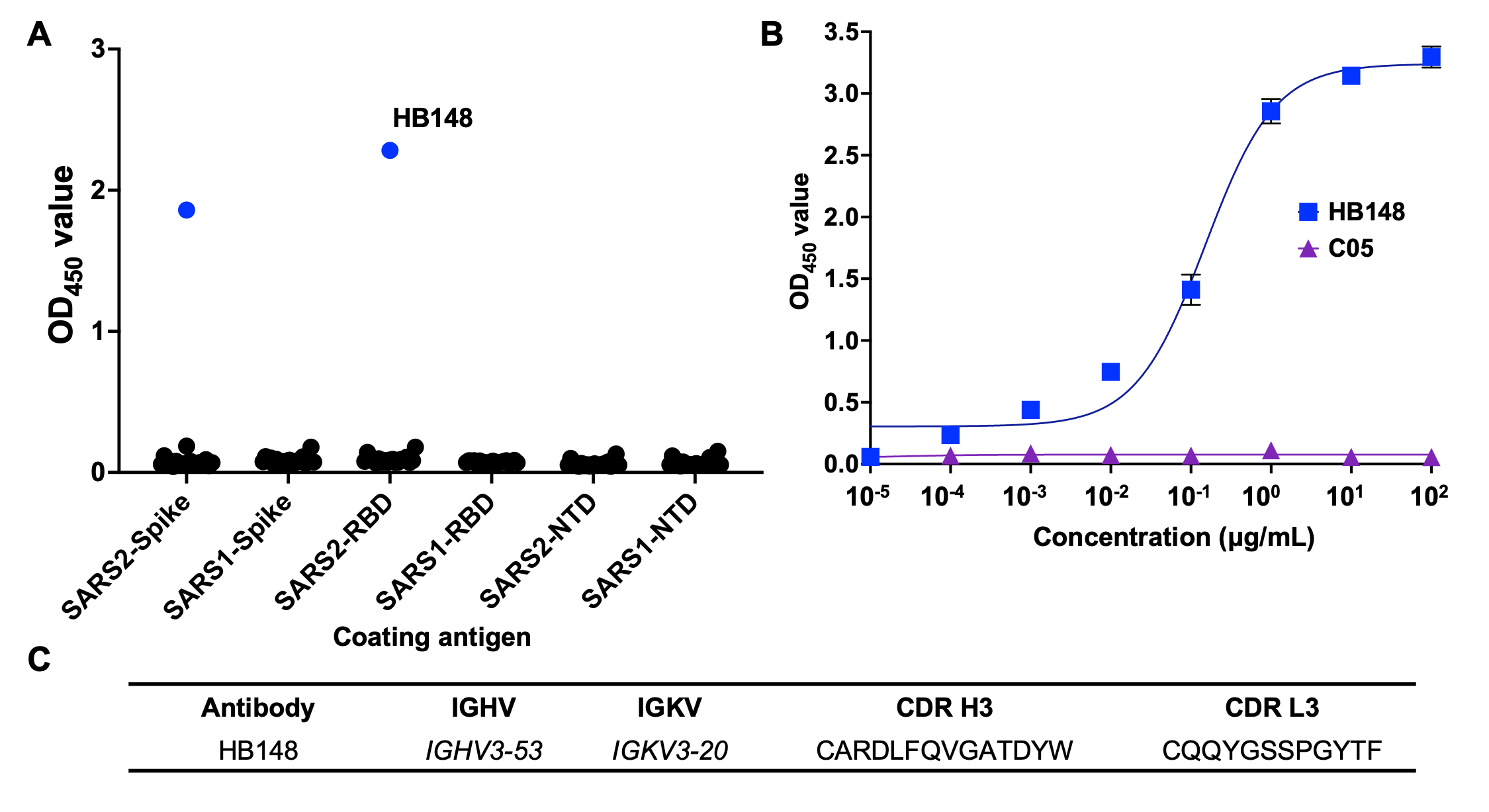

Supplement: Supplementary file 2 — Supporting File 2: advs76522‐sup‐0002‐FigureS1‐S13.zip. [file ADVS-9999-e76522-s001.zip › Figure S1_Phenotype.png]

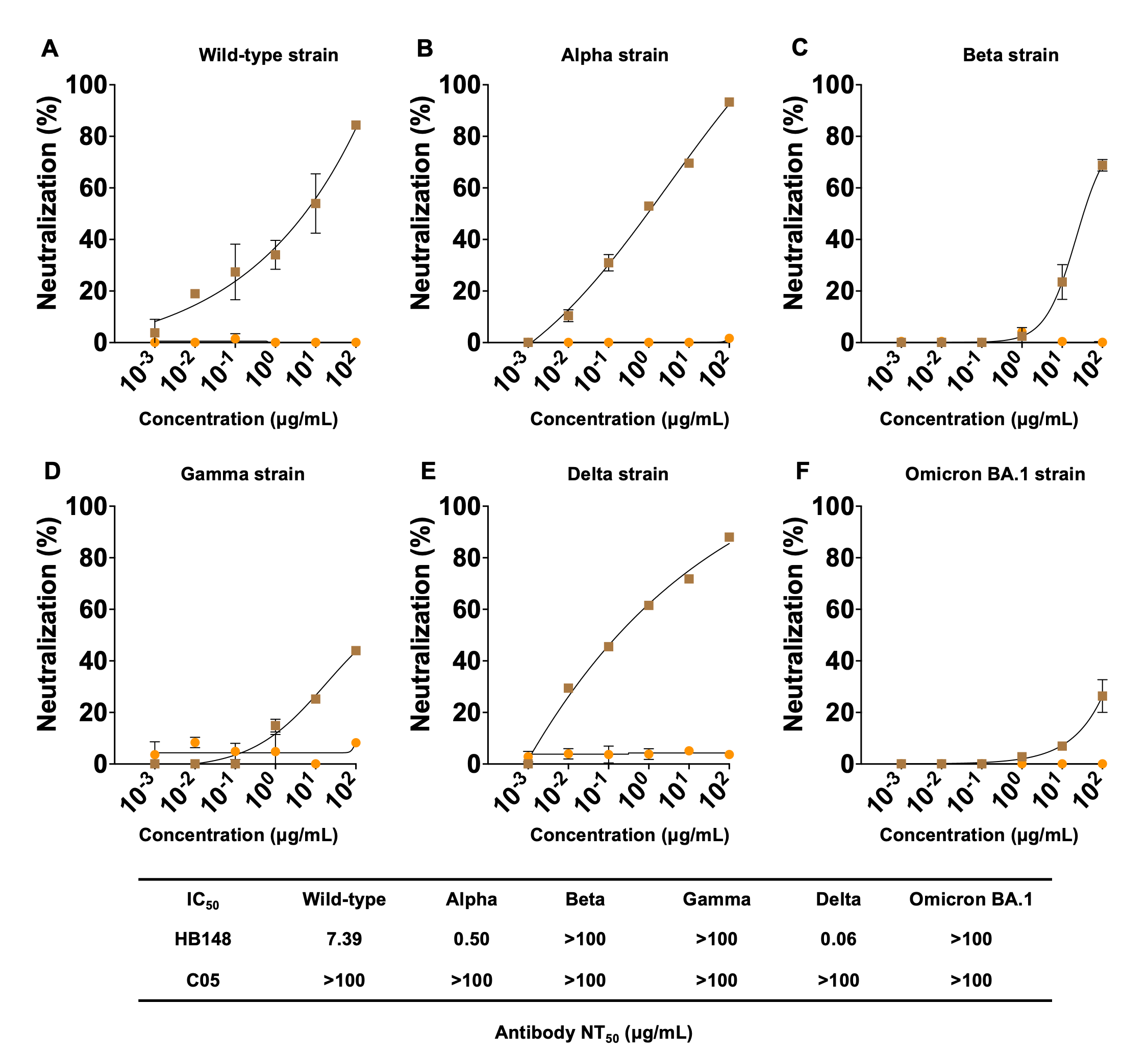

Supplement: Supplementary file 2 — Supporting File 2: advs76522‐sup‐0002‐FigureS1‐S13.zip. [file ADVS-9999-e76522-s001.zip › Figure S2_pseudovirus neutralization-Lv2.png]

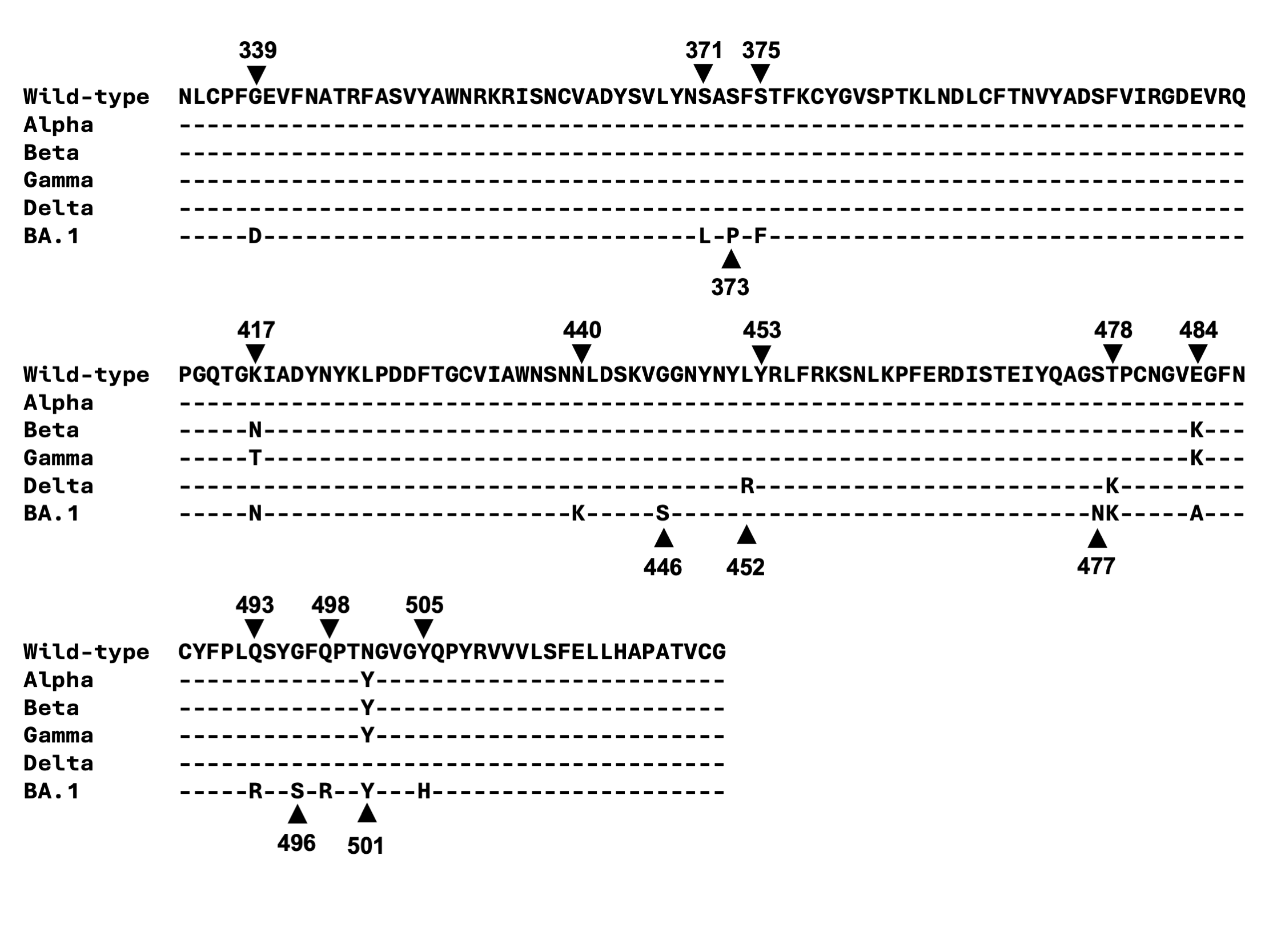

Supplement: Supplementary file 2 — Supporting File 2: advs76522‐sup‐0002‐FigureS1‐S13.zip. [file ADVS-9999-e76522-s001.zip › Figure S3_Sequence alignment of variants-Lv1.png]

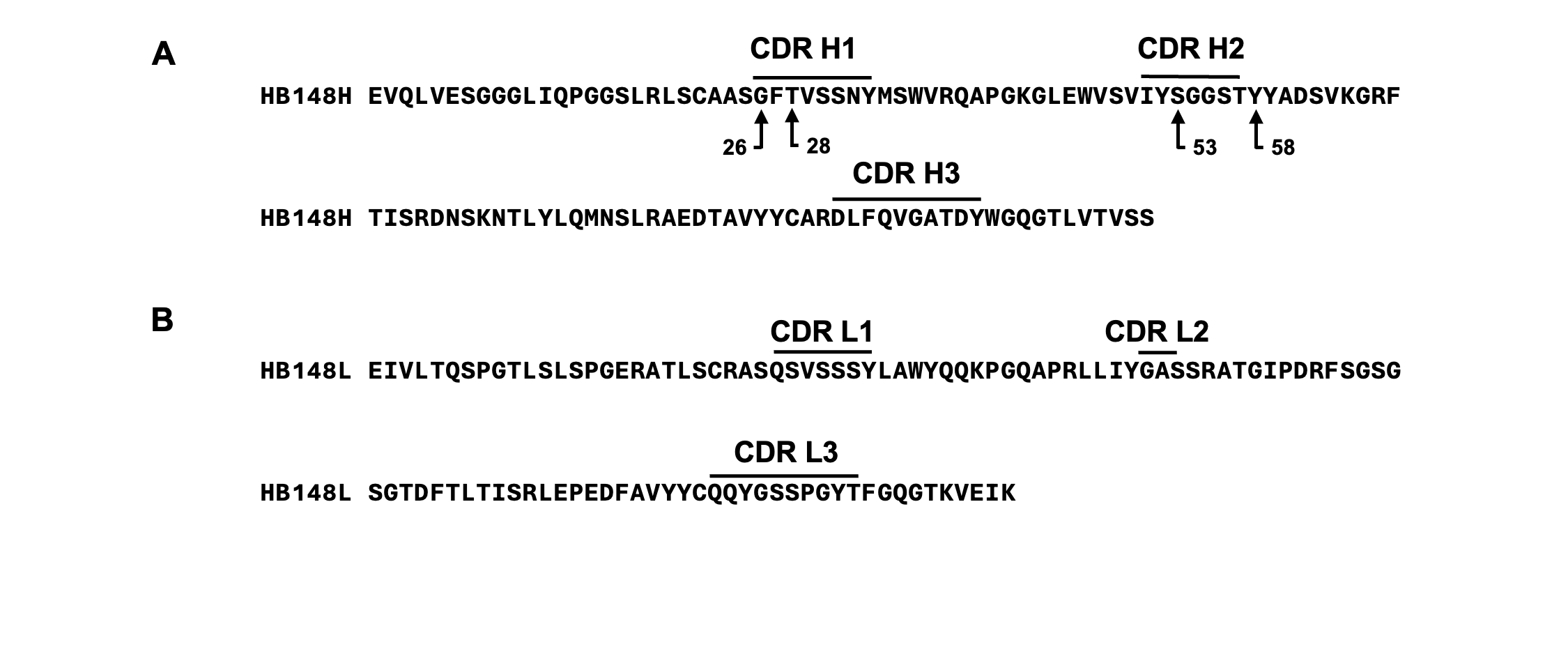

Supplement: Supplementary file 2 — Supporting File 2: advs76522‐sup‐0002‐FigureS1‐S13.zip. [file ADVS-9999-e76522-s001.zip › Figure S4_HB148 SHM.png]

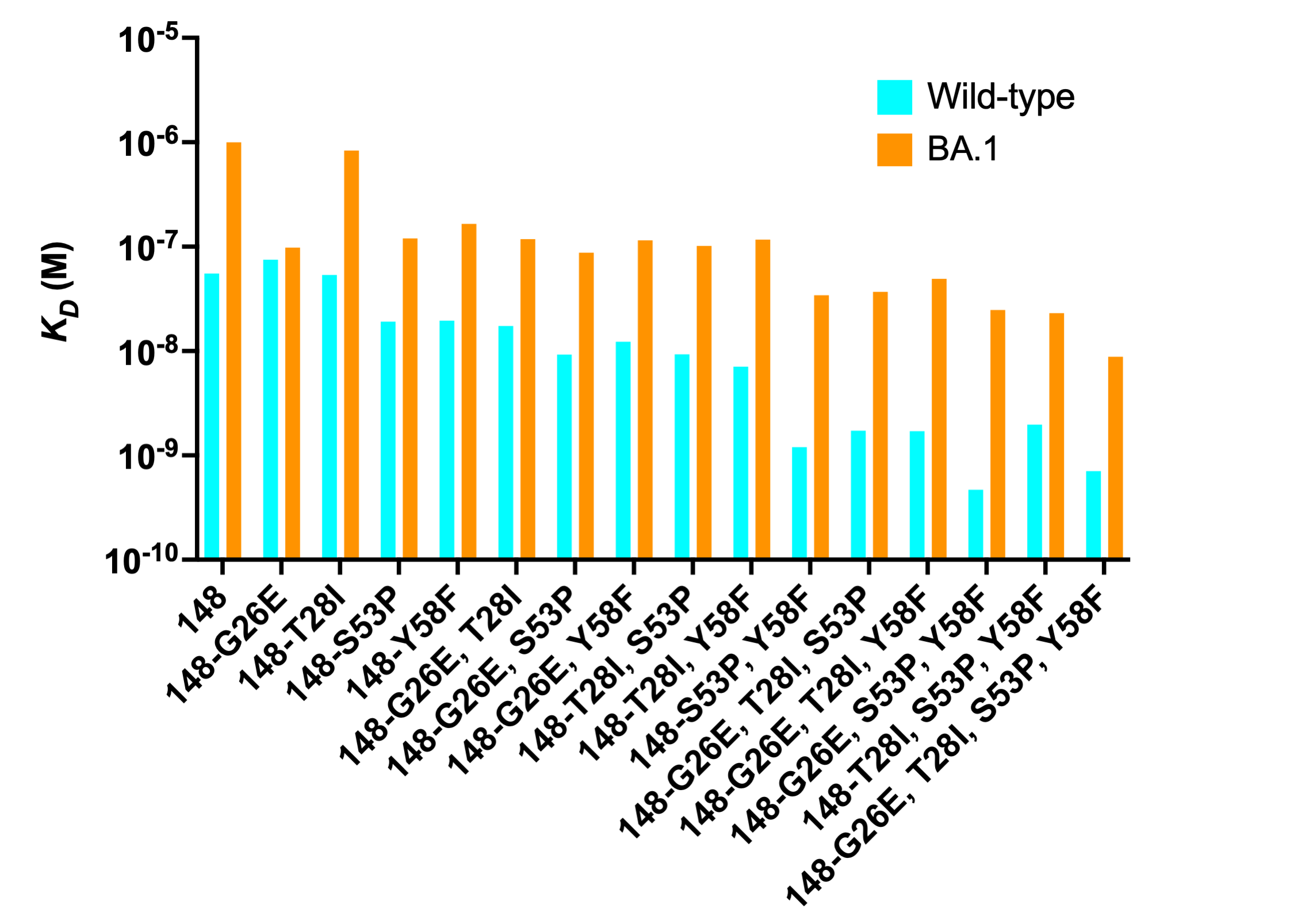

Supplement: Supplementary file 2 — Supporting File 2: advs76522‐sup‐0002‐FigureS1‐S13.zip. [file ADVS-9999-e76522-s001.zip › Figure S5_BLI data for single mutations.png]

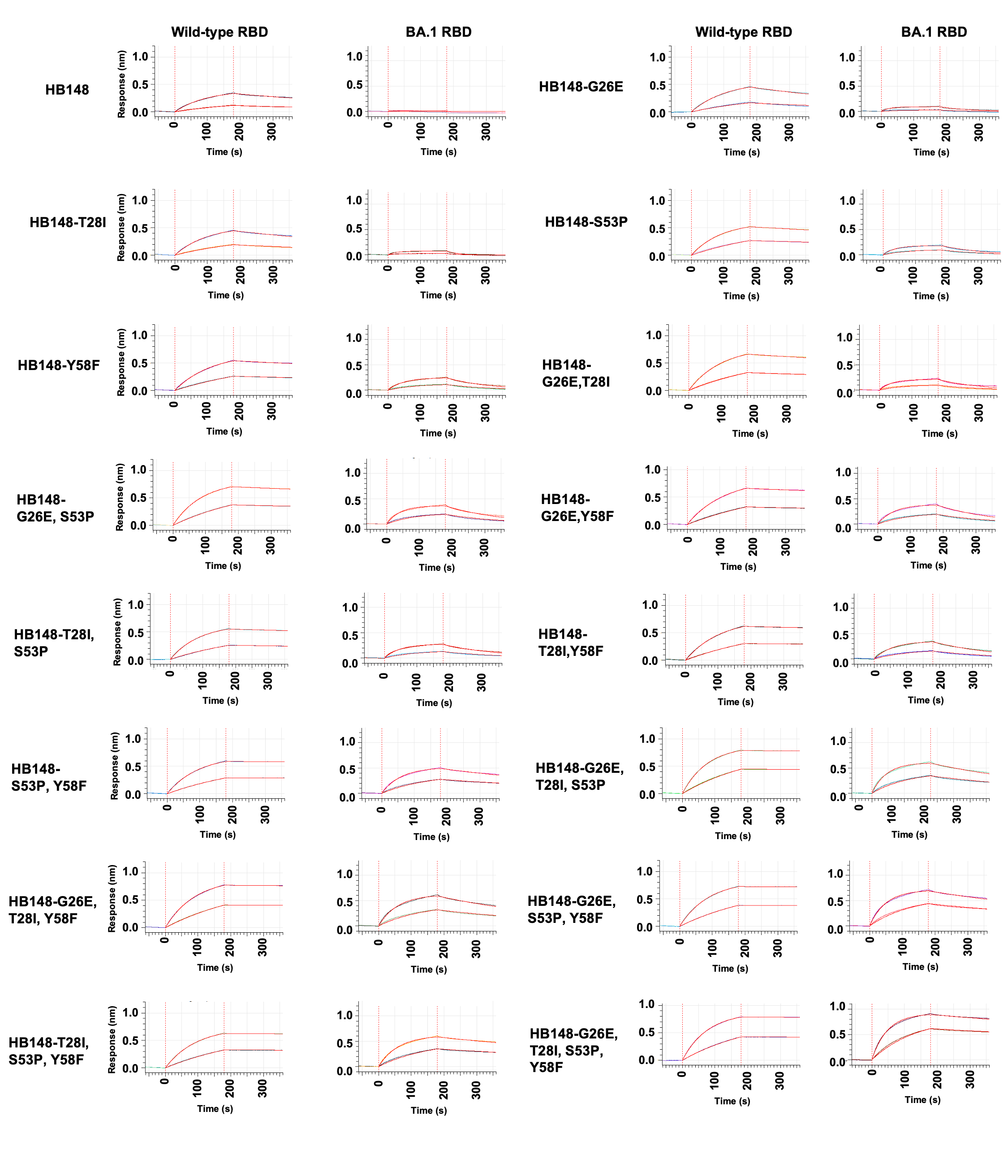

Supplement: Supplementary file 2 — Supporting File 2: advs76522‐sup‐0002‐FigureS1‐S13.zip. [file ADVS-9999-e76522-s001.zip › Figure S6-BLI-148.png]

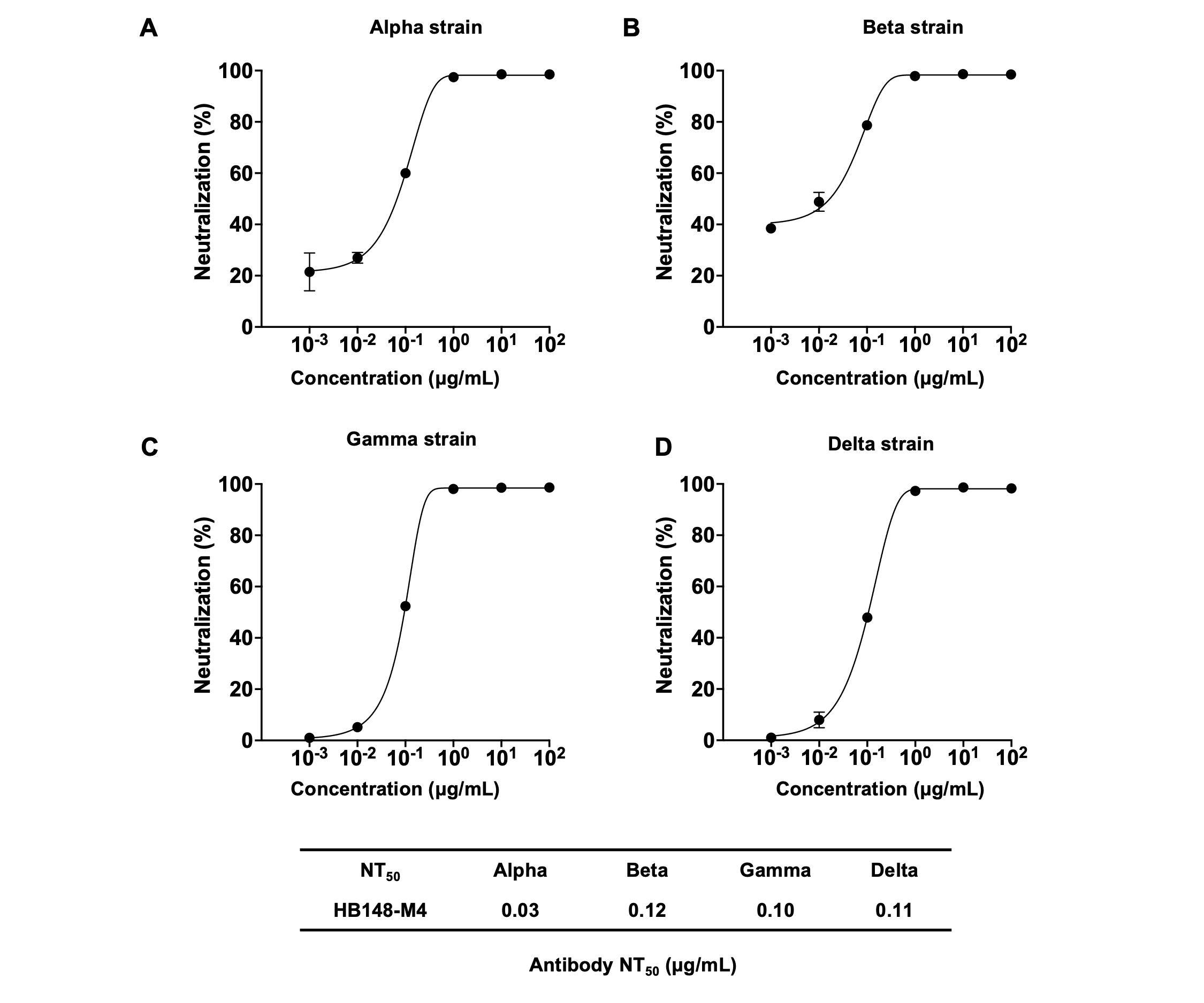

Supplement: Supplementary file 2 — Supporting File 2: advs76522‐sup‐0002‐FigureS1‐S13.zip. [file ADVS-9999-e76522-s001.zip › Figure S7_HB148-M4-svnt neutralization.png]

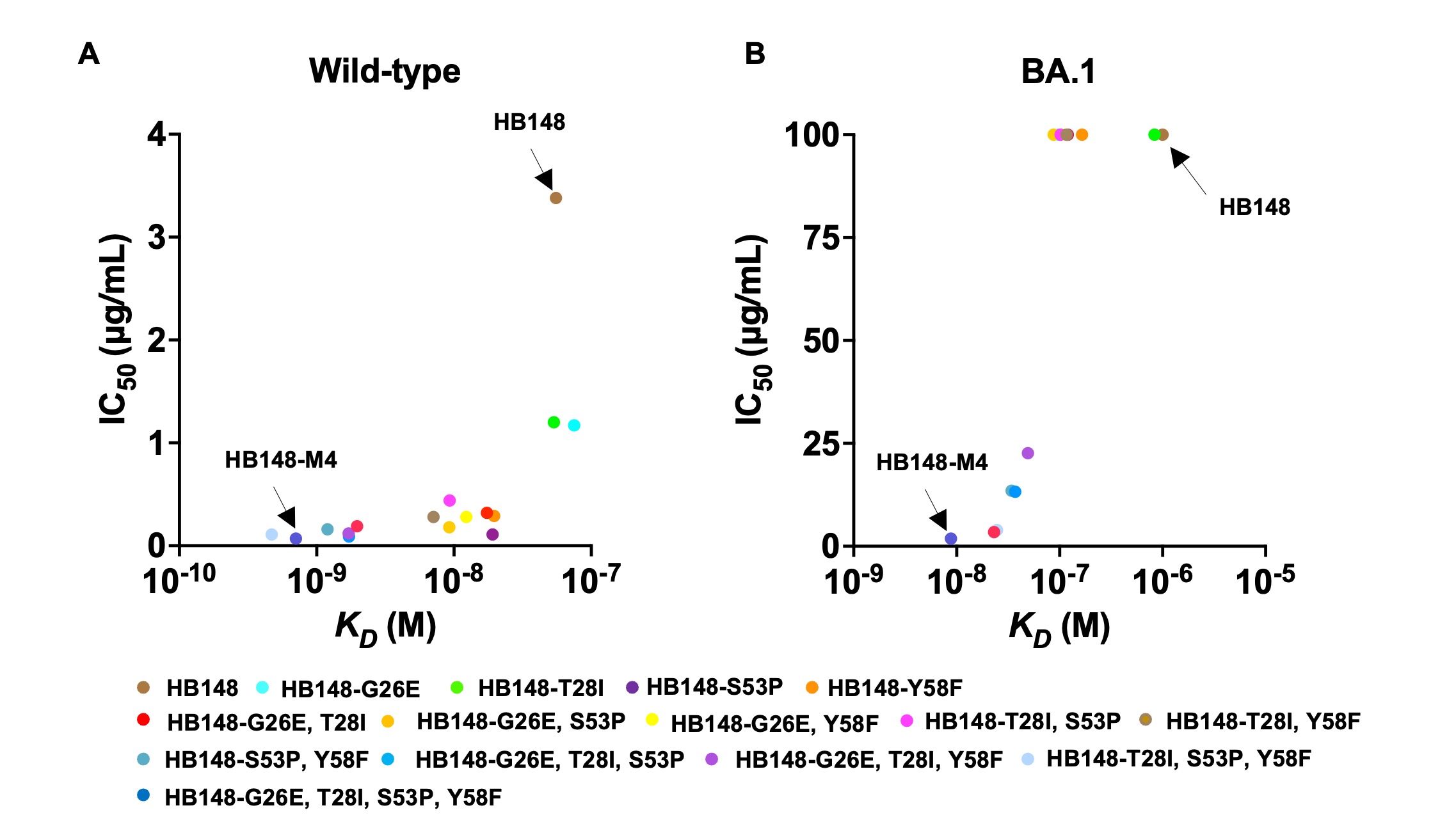

Supplement: Supplementary file 2 — Supporting File 2: advs76522‐sup‐0002‐FigureS1‐S13.zip. [file ADVS-9999-e76522-s001.zip › Figure S8_Correlation between BLI and sVNT.jpg]

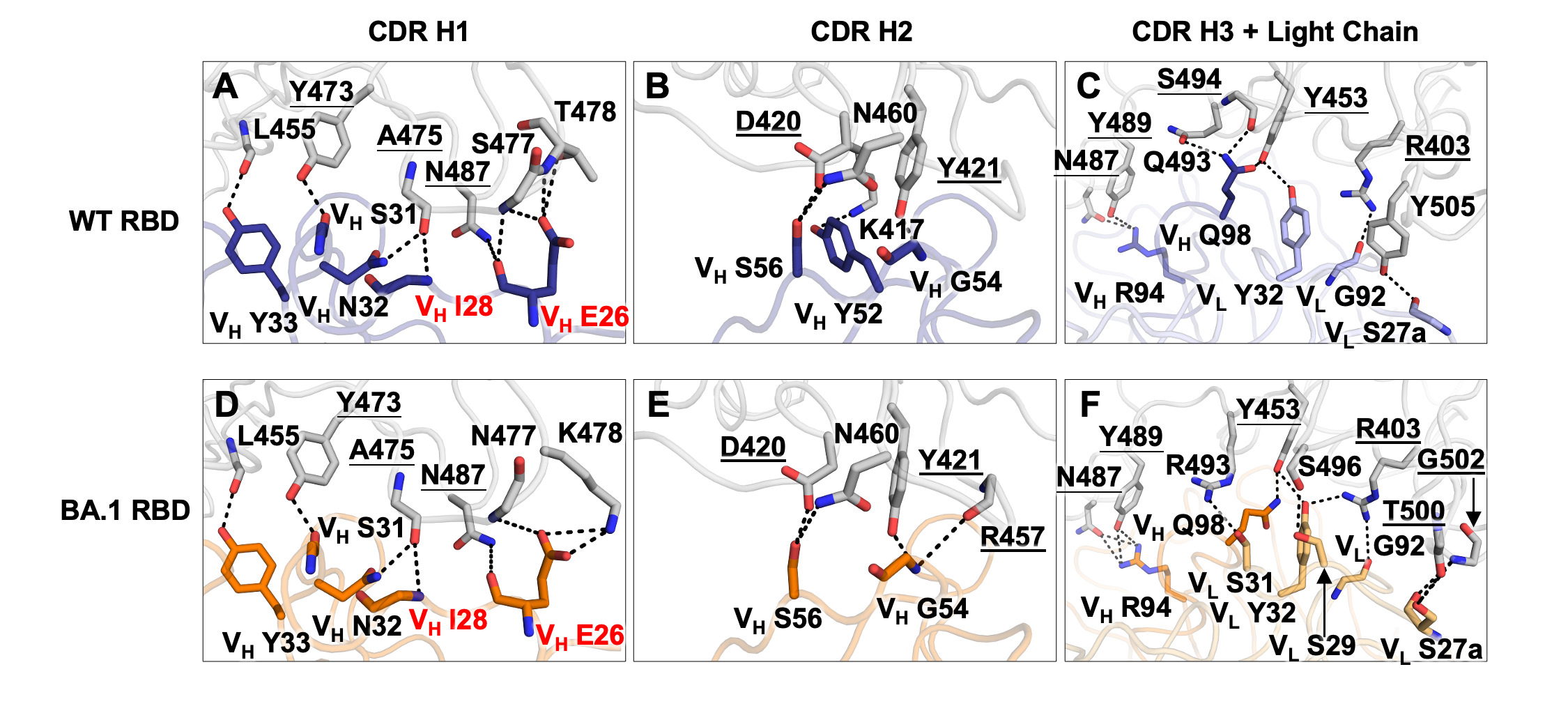

Supplement: Supplementary file 2 — Supporting File 2: advs76522‐sup‐0002‐FigureS1‐S13.zip. [file ADVS-9999-e76522-s001.zip › Figure S9_WT-RBD with HB148-M4.png]
